# Defining Power and Agency in Gender Relations in El Salvador: Consequences for Intimate Partner Violence and Women’s Mental Health

**DOI:** 10.3389/fpsyg.2022.867945

**Published:** 2022-04-19

**Authors:** Laura Navarro-Mantas, Soledad de Lemus, Efraín García-Sánchez, Lucy McGill, Nina Hansen, Jesús L. Megías

**Affiliations:** ^1^Mind, Brain and Behaviour Research Centre, University of Granada (CIMCYC-UGR), Granada, Spain; ^2^Department of Social Psychology, Institute of Psychology, University of Groningen, Groningen, Netherlands

**Keywords:** violence against women, intimate partner violence, mental health, agency, power, Global South

## Abstract

Intimate partner violence (IPV) affects thousands of women around the world and is prevalent in the Global South. Unequal social structures perpetuate hierarchies and maintain women’s vulnerability to violence. Difficulties women face in accessing education, economic resources, and employment diminish their power in intimate relationships, increasing the likelihood of IPV. These factors can also have a significant effect on women’s mental health. However, some studies show that economic empowerment does not necessarily translate into greater agency for women if they cannot use the resources they earn to pursue whatever goals or values they regard as important in life. Agency is women’s ability to identify their life goals and act upon them through critical evaluation (intrinsic agency) and autonomous decision-making (instrumental agency). In this article, we aim to analyze the relationship between women’s power (educational and economic) and agency and their influence on intimate partner violence and on women’s mental health in the context of El Salvador. Currently, El Salvador has one of the highest percentages of femicide worldwide. We used data from the first national survey on violence against women in El Salvador to determine empowerment indicators and investigated their influence on intimate partner violence and women’s mental health. Results from a representative sample of 1,274 women aged between 15 and 64 years old and, using a structural equation modeling revealed that education was a protective factor against IPV, but economic power appeared to put women at greater risk of IPV. Education was positively related to both intrinsic and instrumental agency, but only instrumental agency was negatively associated with the likelihood of being a victim of IPV. Finally, both intrinsic and instrumental agencies were positively related to women’s mental health. We discuss the importance of identifying specific factors related to women’s power and agency to prevent IPV and mental health problems and to promote more gender equity in the Global South.

## Introduction

The World Health Organization (WHO) and the United Nations (UN) rank gender justice as one of today’s most urgent goals. Violence against women is recognized as a severe human rights violation, meaning that women’s rights to the health and safety of their bodies are at the top of the global political agenda (United Nations [UN], 1994). One of the seventeen sustainable development goals identified by the United Nations (UN General Assembly, 2015), has been achieving gender equity and empowering all women and girls, especially in the countries of the Global South. Many interventions aim to strengthen the position of women around the world. However, recent global data still show that 26% of women aged 15 and older who have ever been in an intimate relationship have been subjected to some form of physical or sexual violence by their partner (World Health Organization [WHO], 2021; Sardinha et al., 2022). Intimate partner violence (IPV) can take the form of physical, sexual, emotional, or controlling violence (United Nations [UN], 1994), and all of these violence types have a negative impact on women’s mental health (Navarro-Mantas et al., 2021a). The current study aims to investigate how women’s empowerment (in terms of their power to access education and economic resources, and their levels of agency) is related to IPV and mental health and to offer a systematic analysis of the risk and protective factors of violent victimization in a country, like El Salvador, with one of the highest rates of femicide in the Global South.

### Gendered Power and Intimate Partner Violence

The United Nations [UN] (1994) affirms that the violence suffered by women, both in the lower and higher-income nations, is related to inequality of power. Power is rarely evenly divided and is often disproportionately awarded to men instead of women (Pratto and Walker, 2004). Power inequalities influence IPV against women (Malik and Lindahl, 1998). Furthermore, the structural base of gender inequities and related cultural norms can increase women’s risk of experiencing IPV (Connell, 2014; Dutt et al., 2016). Therefore, IPV is often rooted in the structure of marriage, family, and community in which women are situated. Women’s limited access to education, economic resources, and employment diminishes their power in intimate relationships, which increases the probability of their being subjected to violence (Dobash and Dobash, 1979; Pratto and Walker, 2004). Specifically, having children (Ali et al., 2013; Navarro-Mantas et al., 2021b), being younger (Bauer et al., 2000; McPherson et al., 2007), being divorced or separated (Smith et al., 2002), having low economic resources (Ellsberg et al., 1999), living in rural or peripheral areas (Loxton et al., 2006), and having a low education level (Navarro-Mantas et al., 2021b), have been documented as risk factors for IPV. Additionally, in societies in which violence may be socially accepted or prevalent, as in El Salvador, the risk of experiencing IPV is greater (Jewkes, 2002). Many of these sociodemographic characteristics, which are the consequences of gender inequality, also have a fundamental impact on women’s mental health and well-being (Ludermir et al., 2008; Navarro-Mantas et al., 2021a).

Intimate partner violence affects women worldwide, regardless of their socioeconomic status and educational level. However, the prevalence of IPV is higher in low-income and middle-income countries and regions (Sardinha et al., 2022). The increased vulnerability of women in countries in the Global South is associated with the different social, economic, and political circumstances that limit women’s ability to leave abusive relationships, such as economic vulnerability, inequitable gender norms, prejudice and gender stigma, discriminatory law, weak support services, and social conflict (World Health Organization [WHO], 2021; Sardinha et al., 2022). To reduce and prevent violence against women, systematic research is needed to better understand how structural inequities may perpetuate a system of power imbalances (Grabe, 2010; Else-Quest and Grabe, 2012).

High levels of female empowerment can protect women against IPV; however, to date, there is little consensus on how to best empower women as the societal power inequities women are embedded in may differ among cultural contexts (Jewkes, 2002). Empowerment can be achieved by women’s access to material (e.g., money and properties), social (e.g., social status and respect), human (e.g., education), and environmental (e.g., violence-free contexts) resources (Kabeer, 1999; Malhotra and Schuler, 2005). A recent review of interventions to prevent violence against women concluded that interventions addressing economic empowerment effectively reduced physical and sexual IPV (Kerr-Wilson et al., 2020). Similarly, another review showed that economic interventions may have the largest effects on women’s empowerment in fragile nations in the Global South; however, gender and social norms acted as significant barriers for women (Lwamba et al., 2021). Thus, it is fundamental to identify the barriers and the underlying processes that prevent women’s empowerment. Dutt and Grabe (2017) reviewed various ways in which the processes of social transformation toward greater empowerment was hindered. For example, women face obstacles participating in different social areas, such as political and community decisions, due to their family responsibilities or patriarchal gender roles which define clear and separated spaces for women and men. Furthermore, women’s empowerment is hindered when public officials or other personnel involved, merely display a narrative of women’s “salvation” but do not share a commitment for transformation and social justice (Grabe, 2015). At the same time, women have limitations in accessing opportunities which prevents the development of their leadership and decision-making skills (Lachance-Grzela and Bouchard, 2010; Forste and Fox, 2012). Therefore, interventions aimed at empowering women require larger social transformations than solely access to economic resources.

### Defining *power to* and *power over*

In social psychology, several authors have studied the role of power in IPV (Gage and Hutchinson, 2006; Megías and Montañés, 2012; Grose and Grabe, 2014). Grose and Grabe (2014) distinguished between two types of power. First, *power to* refers to levels of social, material, and human resources, such as access to education or employment. These give women the ability to actively participate in society, pursue their interests, and act upon their wishes to achieve their goals. Second, *power over* implies the ability to influence the behavior of relevant others, such as an intimate partner.

In studies on IPV against women, women’s age, income, educational level, and occupational status are commonly used as *power to* measures since these resources can reduce economic or social dependency on their partner and enhance women’s ability to control their boundaries, resist violence. or escape abuse (Oropesa, 1997; Grose and Grabe, 2014). In general, women with higher education seem to be at lower risk of IPV (e.g., Karamagi et al., 2006; Vung et al., 2008; Gass et al., 2011; Conroy, 2014). Also, having more economic power may be associated with lower incidences of IPV (Schuler et al., 1996; Bhattacharya et al., 2011). However, a systematic review that analyzed interventions seeking to increase the economic power of women in low-income nations drew inconclusive results: participation in women’s economic empowerment groups had statistically significant positive effects on several dimensions of women’s empowerment (economic, social, and political) but not on the psychological empowerment (Brody et al., 2015). It appeared that the positive impact of these groups was from empowerment in skills, such as knowledge about economic management, independence in decision-making, autonomy, and social support. Conversely, other scholars argue that economic empowerment can also put women at greater risk of suffering IPV (Ludermir et al., 2008; Nguyen et al., 2008; Rahman et al., 2011; Kurtiş et al., 2016; Bulte and Lensink, 2019). From this perspective, women with greater control of resources than their male partners would be exposed to a greater risk of violence, by challenging the male status of the main family provider. Indeed, some men may exercise IPV to regain greater control caused by this perceived break from traditional gender roles (for a more detailed discussion, refer to Dutt et al., 2016; Huis et al., 2019). Stimulating economic participation may empower women in countries of the Global North but may cause potential harm in the Global South (e.g., Kurtiş et al., 2016). Developed and well-intended in the North but implemented in the South, such interventions may deprive people of their traditionally developed connections and may reproduce historical and ongoing forms of (neo)colonial domination (Kurtiş et al., 2016). The current study aims to understand and investigate the role of different aspects of *power to* in explaining IPV against women and women’s mental health.

Researchers have used several indicators in an attempt to approximate *power over*. Common indicators used include an evaluation of marital differences in the contribution of resources, differences in the control over household decision-making, and the couple’s control over the behavior and mobility of their spouse (Grose and Grabe, 2014). Research in Latin American contexts may offer evidence of the influence of *power over* dynamics on women’s empowerment. For example, results of a national survey in Colombia showed that decision-making within the relationship (a clear indicator of *power over*), was influenced by continuous negotiation processes within the family about topics, such as money, education, health, and use of time, which are affected by gender power differences (Friedemann-Sánchez and Lovatón, 2012). Being the main breadwinner does not always translate to having greater decision-making power for women in this context as they are responsible for the family (Yount et al., 2021). Therefore, having financial responsibilities might mean greater stress and lower levels of well-being. Gendered power is complex, and we first must critically reflect from a cultural and gender perspective on what power means for women in specific Latin American cultural contexts.

### Power and Agency as Part of Empowerment

Empowerment is a multifaceted process (Huis et al., 2019) and a large body of research has investigated the role of women’s empowerment in relation to violence against women (Vyas and Watts, 2009; Eggers del Campo and Steinert, 2020). Women’s empowerment is defined as women’s ability to make strategic life choices in settings in which this ability was previously denied to them; this includes life choices in relation to resources, agency, and achievements (Kabeer, 1999). Women’s ability to increase their life options beyond the resources available to them is fundamental for empowerment (Richardson et al., 2019; Yount et al., 2021). Agency is defined as a person’s freedom and capacity to make decisions about their own life in accordance with personal goals (Sen, 1985). It entails that women have the ability to increase their life choices, identify their objectives, and act upon them, and is considered the central component of the broader concept of women’s empowerment (Kabeer, 1999; Malhotra and Schuler, 2005). Agency includes internal qualities, such as critical thinking (Mosedale, 2005) corresponding to what has been termed “intrinsic agency” (Miedema et al., 2018) and the ability to make decisions as externally observable factors known as “instrumental agency” (Kabeer, 1999; Miedema et al., 2018).

Studies have shown that a positive relationship exists between women’s access to material (e.g., wealth), social (e.g., social status and respect), human (e.g., education) and environmental resources (e.g., contexts free of violence), and their agency (Kabeer, 1999; Malhotra and Schuler, 2005). A recent literature review^1^ showed that access to microfinance services was associated with (1) increased levels of personal empowerment (referring to an individual’s personal beliefs and actions), and (2) mixed results for relational empowerment (referring to beliefs as well as actions in relation to relevant others, such as their intimate partner) among female entrepreneurs (Huis et al., 2019). Financially empowering measures, such as microfinance services, have been shown to positively affect the instrumental agency and family decisions, such as freedom of movement and contraceptive use (Brody et al., 2015). Furthermore, microcredit programs for women in Bangladesh, improved their autonomy, self-confidence, expression of their opinions and their mobility, their instrumental use of economical services, their decision-making power in marital relationships, and their freedom of movement outside the home (Yount et al., 2021). At the same time, other studies report less positive empowerment and agency effects. For example, gender-bias attitudes, which correspond to the low intrinsic agency, were associated with higher levels of male-controlled sexual decision-making, rape, unprotected sex with a non-primary partner, intergenerational sex, and multiple/concurrent sexual partners in a population study in Botswana and Eswatini (Shannon et al., 2012). Similarly, women’s lack of decision-making power in sexual relationships has been found to be associated with inconsistent condom use (Pettifor et al., 2004) and incident HIV infection (Jewkes et al., 2010) in South Africa, while increased relationship power among women has been associated with lower levels of sexual violence, greater condom use, and greater knowledge of their partner’s serostatus in Kenya (Pulerwitz et al., 2018). To conclude, the reported studies suggest some positive effects on the personal aspects of agency (e.g., self-confidence) but also mixed results with respect to instrumental agency (for example, decision-making and sexual behavior) illustrating the complexity of relational power dynamics. Furthermore, social and cultural gender norms have been identified as major barriers for women’s empowerment in many studies (Jewkes, 2002; Heise, 2011; Zapata-Calvente et al., 2019; Hansen et al., 2021; Lwamba et al., 2021).

### The Cultural Context in El Salvador

Women are especially vulnerable in El Salvador, a country in the Central American region where endemic violence is a structural problem shared by neighboring countries and where traditional gender norms are prevalent (Hume, 2008). The youth gangs (maras) that have taken control of certain areas exercise unprecedented violence over women as part of their domination (Navarro-Mantas et al., 2021a,b). This type of violence is based on extreme control over women’s lives and bodies within the community. Indeed, 54.3% of Salvadoran women have suffered some type of violence (physical, sexual, or psychological) throughout their lives at the hands of a current or former intimate partner (Navarro-Mantas et al., 2021b).

Salvadoran women mainly work in the informal sector and, thus, have less access to social security and, on average, receive a lower income, which reduces their development opportunities (United Nations Development Programme [UNDP], 2020b). Women are the heads in 31.1% of Salvadoran households, which are also generally single parent. The majority of these female-headed, single-parent households in El Salvador have dependents between the ages of 0 and 17. Additionally, 74% of the households face between one and three factors of greater vulnerability: overcrowding, lack of access to drinking water, or informal employment. Furthermore, Salvadoran women spend many hours doing household chores. A recent analysis of the first national survey of violence against women in El Salvador identified clear risk factors for women to experience different forms of violence throughout life (Navarro-Mantas et al., 2021b). Specifically, having children was identified as a risk factor for physical, sexual, and psychological (emotional and controlling) violence. Childcare depends mainly on women, which makes it more difficult for them to access economic resources and employment, reducing their power in their intimate relationships and making violence more likely. Other sociodemographic characteristics and victimization experiences were also related to women’s mental health, especially those associated with gender vulnerability, less power, or fewer resources. For example, older age, lower educational level, and living in a rural area were factors associated with a higher likelihood of mental health problems, regardless of IPV (Navarro-Mantas et al., 2021a). This reality highlights the importance of identifying structural factors related to women’s autonomy and power that help prevent violence in intimate relationships and mental health problems. This study aims to deepen the understanding of the antecedents of IPV against women in El Salvador by analyzing the influence of women’s *power to* and agency on violence victimization and mental health.

### The Current Study

The current study aimed to offer a more in-depth analysis of the link between aspects of women’s *power to* and agency, their likelihood of experiencing IPV, and their mental health in a representative sample of Salvadoran women. More precisely, we operationalized *power to* as women’s access to education and economic resources (including whether women have a job and whether they have ownership of certain assets). We investigated three hypotheses. Based on a previous study (Grose and Grabe, 2014), we, first, expected that women who have more *power to* (that is higher levels of education and more access to economic possibilities and assets) would be less likely to be subjected to IPV. Second, we hypothesized that women who have more *power to* should have better mental health, as they may have more access to resources in their communities. Third, we hypothesized that intimate partner violence would mediate the relationship between women’s *power to* and their mental health.

In addition, we explored the mediating role of agency (intrinsic and instrumental) in the relationships between *power to*, IPV, and mental health. We expected that *power to* should influence both intrinsic agency (women’s understanding of their rights, sense of gender equality, and justification of IPV) and instrumental agency (that is their ability to make decisions that impact their life, social support, and freedom of movement), which in turn will impact their likelihood of being subjected to IPV and their mental health.

## Materials and Methods

### Study Design and Participants

We used data from a cross-sectional national survey conducted in El Salvador between December 2013 and February 2014 as a part of the WHO multi-country study (Navarro-Mantas et al., 2021a,b). Data were collected by using a systematic random probabilistic sampling method by household. One woman from each household was randomly selected to be interviewed. The total sample size consisted of 1,521 women from rural and urban areas across the 14 administrative divisions and 262 municipalities of El Salvador. We selected participants who have had an intimate partner, to assess the prevalence of violence against women by an intimate partner (World Health Organization [WHO], 2004). For quality control purposes, only those interviews which were 80% completed and where the most essential sections were answered (that is, sections on violence and health) were included in the final data set. In total, 16.23% of interviews which did not fulfill these criteria were excluded. The final sample was composed of 1,089 women (*M* = 38.27 years, *SD* = 13.14, range: 15–64). The rate of refusal to participate in the survey was 4.23%. For more information on the sampling procedure, refer to Navarro-Mantas et al. (2021b).

### Procedure

The data were collected according to the World Health Organization [WHO] (2004) protocols with regard to the implementation stages and appropriate management of methodological and ethical issues. The interviews were conducted in private places without the presence of children (Garcia-Moreno et al., 2006), and they lasted for about 60 min. All participants gave informed consent prior to participating. Participation was voluntary and women did not receive any compensation. All members of the research team were women who had completed 3 weeks of specialized training prior to data collection (Jansen et al., 2004). Interviewers also had access to psychological care groups for emotional support. The study design was approved by the National Clinical Research Ethics Committee of El Salvador and the Ethics Review Committee of the Pan American Health Organization (PAHOERC) and met the Declaration of Helsinki (2000) criteria. The team used version 11 of the questionnaire designed for the WHO Multi-Country Study on Women’s Health and Domestic Violence against Women (World Health Organization [WHO], 2004). The questionnaire used was in compliance with WHO’s ethical and methodological guidelines for researching IPV (Ellsberg and Heise, 2007). This standardized and structured questionnaire was designed for a Salvadoran population and some minor additions and changes to wording were made in consultation with the WHO/Pan American Health Organization (PAHO) advisory group and consultative committee. The survey was pilot-tested with 50 women. After giving informed consent, women answered questions about their household, demographic information, their community, health, information about their partners, incidences, severity and type of intimate partner violence, the impact of the violence, their coping strategies, and their financial autonomy. Afterward, women were carefully debriefed and thanked.

### Measures

All the measures used were from version 11 of the questionnaire designed for the WHO Multi-Country Study on Women’s Health and Domestic Violence against Women (World Health Organization [WHO], 2004). The wording of the indicators along with descriptive statistics are shown in Supplementary Table 1.

#### Intimate Partner Violence

This measures whether women were subjected to any form of violence by an intimate partner over their lifetime. We operationalized IPV as a second-order latent variable composed of four factors: physical, sexual, emotional, and controlling violence. Indicators were dichotomous such that 1 indicates whether the person was a victim of IPV (1 = “Yes”, 0 = “No”). Women were asked a series of questions related to whether they had ever experienced a range of behaviors relating to one of these four categories.

#### Mental Health

We operationalized mental health as a latent variable composed of nine self-reported indicators about having symptoms of psychological distress. The indicators are part of the Self-Report Questionnaire–20 (SRQ-20, Harding et al., 1983), which includes 20 questions about somatic, anxious, and depressive symptoms. This questionnaire assesses symptoms of common mental disorders in the context of primary health services, such as somatoform disorders, depression, and anxiety with symptoms of insomnia, fatigue, irritability, poor memory/concentration, and somatic complaints, including headaches, trembling, or indigestion (Goldberg and Huxley, 1992), in the 4 weeks prior to the evaluation. We conducted an EFA on the 20 items and selected the items that were loaded in the dominant factor to reduce model complexity. Since some items were loaded onto different factors and had high correlation among them, this operationalization helps the model to reach proper convergence and facilitates substantive interpretation. The indicators were dichotomous (1 = “Yes”, 0 = “No”), but we recoded this variable in such a way that 1 indicates the absence of symptoms or better mental health.

#### Power to

Based on previous literature, *power to* refers to women’s capacity to control or gain access to resources (Grose and Grabe, 2014). In our study, we distinguished between economic and human resources (Kabeer, 1999). We operationalized *power to* based on economic resources with a summative index of six dichotomous indicators (1 = “Yes”, 0 = “No”): having paid employment, earning money through different activities, and owning certain properties (land, a house, a business, and a car). This summative index could range from 0 to 6. Next, we operationalized *power to* based on human resources, that is women’s access to education, using a 4-point scale (0 = “never attended school”, 1 = “basic education,” 2 = “secondary education,” and 3 = “higher education”). *Power to* was modeled as an observed variable since it represents a formative construct. That is, *power to* is the byproduct of adding up different uncorrelated indicators (e.g., income, properties, etc.) rather than representing an underlying latent factor.

#### Agency

This variable refers to a person’s freedom and capacity to make decisions about their own life in accordance with personal goals (Sen, 1985). This variable comprised both an instrumental dimension related to freedom of movement, decision-making, and social support; and an intrinsic dimension based on personal values and beliefs (Richardson et al., 2019). The instrumental agency was operationalized as a formative variable computed as a summative index of six dichotomous indicators (1 = “Yes,” 0 = “No”) that create the conditions of freedom to participate in different social circles, make reproductive decisions, and engage in supportive relationships (answers could range from 0 to 6). The intrinsic agency was operationalized as a latent variable composed of four dichotomous indicators (1 = “Agree,” 0 = “Disagree”) related to women’s beliefs about intimate partner relationships.

#### Control Variables

We also controlled for the potential influence of sociodemographic variables, such as age (in years), area of residence (1 = “Rural,” 0 = “Urban”), and number of living children.

## Results

### Analytical Approach

We tested our hypothesized model by using the structural equation modeling (SEM) framework. We used confirmatory factor analyses (CFA) to examine the goodness of fit of the measurement model, and then tested the structural part by estimating both direct and indirect effects implied in our model. We used a robust weighted least squares estimator to test our model, as this is appropriate for handling dichotomous indicators, such as ours (Kline, 2011). We used common benchmarks for assessing the goodness of fit of our model, such that fit statistics should be above 0.90 for comparative fit indices (CFIs) and Tucker-Lewis index (TLI), and below 0.06 for RMSEA and SRMR (Hu and Bentler, 1999; Kline, 2011). We reported the robust versions of all the fit statistics.

Given the amount of variables implied in our hypothesized model, we divided our analytical approach into two stages. First, we conducted a confirmatory model in which we tested the predictive effect of our *power to* variables (educational and economic resources) on IPV and mental health. Furthermore, we examined the extent to which the relationship between our *power to* variables and mental health was mediated by the likelihood of being a victim of IPV.

In the second stage, we adopted an exploratory perspective, where we investigated the role of intrinsic and instrumental agency in the previous models. We examined whether intrinsic and instrumental agency were potential mechanisms through which *power to* variables exert their effect on both IPV and mental health. In a full model, including the variables of interest of this study, we tested the mediating role of the agency variables between *power to* and IPV and between *power to* and mental health. This exploratory model deepens our understanding of potential mechanisms through which *power to* variables influence well-being. Since these analyses were exploratory, we did not develop hypotheses in advance. However, based on the literature, we posit that *power to* should influence IPV and mental health by altering women’s levels of agency. Therefore, having more *power to* resources will bring more physical and psychological autonomy (that is, more instrumental and intrinsic agency) which in turn, should reduce the likelihood of being subjected to IPV and should increase the mental health levels.

### Descriptive Analyses

Table 1 shows the correlations between the latent and observed variables included in our study. As can be seen from Table 1, education has a significant positive correlation with mental health (*r* = 0.27, *p* < 0.001), intrinsic agency (*r* = 0.37, *p* < 0.001), and instrumental agency (*r* = 0.15, *p* < 0.001), and has a negative relationship with IPV (*r* = −0.17, *p* < 0.001). Economic power has a significant positive correlation with IPV (*r* = 0.22, *p* < 0.001) but does not strongly correlate with the other variables. Intrinsic (*r* = 0.22, *p* < 0.001) and instrumental agency (*r* = 0.17, *p* < 0.001) both have a significant positive correlation with mental health.

**TABLE 1:** Zero-order correlations between variables (latent and observed) included in the study.

	1	2	3	4	5
(1) Instrumental agency	–				
(2) Mental health	0.17***	–			
(3) IPV	−0.16***	−0.36***	–		
(4) Intrinsic agency	0.08*	0.23***	0.05	–	
(5) Power to (economic)	0.01	0.08	0.22***	0.10*	–
(6) Power to (education)	0.15***	0.27***	−0.17***	0.37***	0.09**

***p < 0.001, **p < 0.01, *p < 0.05.

### Confirmatory Analyses

Before testing our hypotheses, we confirmed that the measurement model provided appropriate fit statistics, which indicated that the latent variables were well represented by the data, χ^2^(2) = 0.920, *p* < 0.631, *CFI* = 1.000, *TLI* = 1.005, RMSEA = 0.001, 90% CI = [0.001, 0.049], SRMR = 0.017.

**FIGURE 1:**
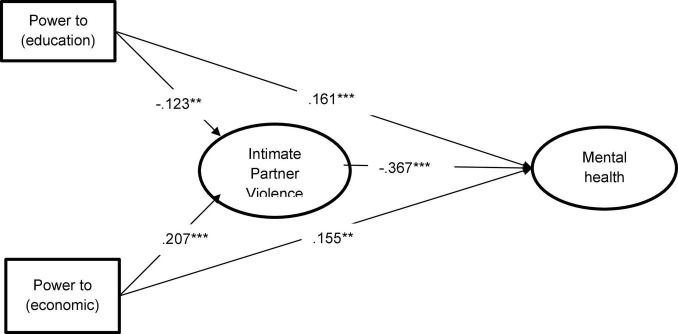
Standardized regression coefficients for the structural regression model of our hypotheses. ***p < 0.001, **p < 0.01; factor loadings and first-order latent variables are omitted for simplifying the interpretation.

Regarding the structural model, we found that our hypothesized model offered appropriate fit statistics, χ^2^(645) = 943.799, *p* < 0.001, CFI = 0.990, TLI = 0.992, RMSEA = 0.024, 90% CI = [0.020, 0.027], SRMR = 0.080. The structural regression coefficients indicated that the likelihood of suffering IPV was negatively predicted by having more education, but it was positively predicted by controlling more economic resources. That is, higher levels of education protected women from being a victim of IPV but accessing more economic resources was related to increased vulnerability to intimate partner violence. Furthermore, as expected, IPV negatively impacted mental health (refer to Figure 1; full information about factor loadings and parameter estimates are available at Supplementary Tables 2–4).

Furthermore, we found an indirect effect from *power to* variables to mental health through IPV. *Power to* (education) was positively related to mental health *via* reduced likelihood of being a victim of IPV (β = 0.045, *SE* = 0.019, *p* = 0.019; 95% CI [0.008, 0.082]. The total effect of *power to* (education) on mental health (sum of the direct and indirect effect) was positive and statistically significant (β = 0.206, *SE* = 0.047, *p* < 0.001, 95% CI [0.113, 0.299].

Conversely, we found that *power to* (economic) negatively predicted mental health through IPV (β = −0.076, *SE* = 0.019, *p* < 0.001, *95% CI* [−0.113, −0.039]. However, the total effect of *power to* (economic) on mental health was not statistically significant, (β = 0.079, *SE* = 0.047, *p* = 0.094, *95% CI* = [−0.013, 0.171]. This finding suggests that the positive direct effect of *power to* (economic) on mental health was suppressed due to the increase in the likelihood of suffering IPV. That is, the positive influence of controlling economic resources on mental health became suppressed by the indirect influence of IPV.

### Exploratory Analyses

In the second stage of analyses, we examined the extent to which the effect of *power to* on IPV and on mental health could be explained by developing different types of agency. As such, we explored the mediating role of intrinsic and instrumental agency on the association between *power to* and both IPV and mental health.

**FIGURE 2:**
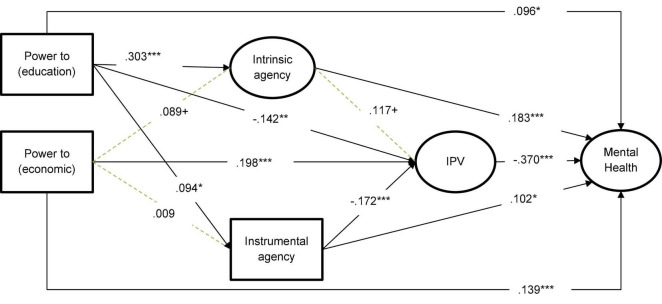
Standardized regression coefficients of an exploratory full model of the mediating role of intrinsic and instrumental agency between *the power to* variables and IPV. *** *p* < 0.001, **p < 0.01; solid indicate statistically significant coefficients and dotted gray lines indicate non-statistically significant coefficients. ^+^*p* < 0.10.

As shown in Figure 2, the intrinsic and instrumental agencies were positively predicted by having more educational resources, but not by controlling economic resources. The association between economic *power to* and instrinsic agency was not statistically significant (*p* = 0.06), nor was it associated with instrumental agency (*p* = 0.77). Furthermore, we found that IPV was reduced by instrumental agency, but the association with intrinsic agency was not statistically significant (*p* = 0.06).

Regarding the roles of intrinsic and instrumental agencies as potential mechanisms for explaining the association between *power to* and IPV, we found that instrumental agency was the only mechanism through which *power to* (education) was negatively related to IPV. That is, the negative effect of *power to* (education) on IPV was partly due to the positive association with the instrumental agency (refer to Table 2).

**TABLE 2:** Standardized indirect effects of Power to (education and economic) on IPV and mental health through the agency (personal and instrumental) variables.

Dependent variable	Predictor and mediator variables	β	*SE*	*p*	95% CI
**IPV**					
	Power-to (economic) → intrinsic agency	0.010	0.008	0.188	[−0.005, 0.026]
	Power-to (economic) → instrumental agency	–0.002	0.005	0.777	[−0.012, 0.009]
	Power-to (education) → intrinsic agency	0.036	0.019	0.066	[−0.002, 0.074]
	Power-to (education) → instrumental agency	−0.016*	0.007	0.030	[−0.031, −0.002]
**Mental health**					
	Power-to (economic) → intrinsic agency	0.016	0.010	0.100	[−0.003, 0.035]
	Power-to (economic) → instrumental agency	0.001	0.003	0.778	[−0.005, 0.007]
	Power-to (education) → intrinsic agency	0.055**	0.019	0.004	[0.019, 0.092]
	Power-to (education) → instrumental agency	0.010	0.005	0.075	[−0.001, 0.020]
	Power-to (economic) → intrinsic agency → IPV	–0.004	0.003	0.197	[−0.010, 0.002]
	Power-to (economic) → instrumental agency → IPV	0.001	0.002	0.777	[−0.003, 0.004]
	Power-to (education) → intrinsic agency → IPV	–0.013	0.007	0.077	[−0.028, 0.001]
	Power-to (education) → instrumental agency → IPV	0.006*	0.003	0.039	[0.000, 0.012]

***p < 0.001, **p < 0.01, *p < 0.05.

With regard to the mediating role of the agency variables in the relationship between *power to* (education and economic) and mental health, we found that only the path from *power to* (education) through the intrinsic agency was statistically significant. That is, *power to* (education) increased intrinsic agency, which in turn was positively related to mental health (refer to Figure 2). Finally, on examining the full model, from *power to* (education and economic) to mental health through agency and IPV, we found that the only statistically significant pathway was the one in which *power to* (education) increased instrumental agency, which in turn reduced IPV, and such a reduction, in turn, improved the mental health (refer to Table 2).

## Discussion

The aim of the current study was to deepen the understanding of power and agency in gender relations in El Salvador, and explore the role of power and agency in women’s likelihood of being victimized as a result of IPV and of suffering from poor mental health. High levels of violence and IPV are present in El Salvador and traditional gender norms are prevalent.

### *Power to* and Intimate Partner Violence

We hypothesized that *power to* based on education and economic access to resources would be negatively associated with IPV which in turn would impact positively on women’s mental health. Results revealed that the power given to women by their education, appeared to protect them from suffering IPV whereas power related to having economic resources appeared to put women at a greater risk of being a victim of IPV. This is in line with previous studies, whereby women who have higher education levels are at reduced risk of IPV (Hadi, 2000; Gass et al., 2011). Education empowers women by increasing their confidence, social contacts, and ability to use the information and other resources in society, which means women are less dependent on their partners (Jewkes, 2002, p. 1,425). Furthermore, our results suggest that economic power puts Salvadoran women at a higher risk of being subjected to IPV. These results coincide with those found in other studies whereby women’s economic power was positively related to their risk of suffering IPV (Casique, 2003; Flake, 2005; Castro et al., 2008; Ludermir et al., 2008; Nguyen et al., 2008; Rahman et al., 2011). For example, a recent study found that across 19 countries in Sub-Saharan Africa, the Middle East, and South Asia, women who were working were significantly more likely to be subjected to IPV than those who were not (Zafar et al., 2021). A possible explanation might be that women who are working could be seen to be violating a prescribed traditional gender norm where women’s traditional role may be perceived to be as a homemaker.

Other studies of interventions developed in Latin America with women and their families have not shown an impact of economic empowerment on IPV (Hidrobo and Fernald, 2013; Bobonis et al., 2020). However, among those that do provide data, it is suggested that economic empowerment is more likely to have a positive impact on the prevention of IPV when it occurs together with other family empowerment strategies, such as intensive education campaigns for some family areas (Hidrobo et al., 2016). In Hispanic/Latino communities the importance of family is emphasized, and women might have more power with regard to familial matters (Marín and Marín, 1991; Malik and Lindahl, 1998). In Mexico, cash transfers that were conditional on children’s education and health checks pointed to a reduction of around 40% in IPV from 2 to 6 years after the intervention, and afterward a monthly money transfer might have allowed many women to leave their partners, although more research is needed for confirmation (Bobonis et al., 2013). In Peru, cash transfer programs linked to children’s education and health resulted in a decrease in physical but not sexual violence against women (Perova, 2010). In Colombia, family protection programs providing money transfers to female heads of household (conditional on children under 7 years accessing health services and those over 7 years attending education), showed a decrease in IPV of at least 5% in participating communities, directly after the money transfer (Camacho and Rodríguez, s.f.; as cited in Kerr-Wilson et al., 2020). In many countries, having children adds to women’s vulnerability (Ali et al., 2013; Navarro-Mantas et al., 2021a). Therefore, accompanying economic empowerment measures with a strengthening of child-rearing support could influence women’s empowerment, their agency, and possibly reduce the likelihood of their being subjected to IPV.

### *Power to* and Women’s Mental Health

Empirical evidence suggests that the impact of IPV on women’s mental health outweighs the influence of other life circumstances and sociodemographic variables (Ludermir et al., 2008; Ishida et al., 2010; Avanci et al., 2013). These findings are consistent with those of our study, whereby *power to* (education and economic resources) had a positive effect on mental health. However, whereas the effect of education on mental health (sum of the direct and indirect effect) was positive and statistically significant, a positive effect for economic resources was not found when women were exposed to IPV.

Our results showed that economic power had a positive effect on women’s mental health, but this effect was neutralized by the greater probability of being subjected to intimate partner violence when women have access to economic resources, and therefore, the overall impact of economic power on mental health was negative. Yount et al. (2021) did not find any relationship between women’s financial power from microfinance programs and their probability of IPV or poor mental health. This difference may be due to economic empowerment gained by participation in microfinance programs that may take longer to have a visible impact. Yount et al. (2021) acknowledged that a greater economic responsibility can coincide with an increase in stress and pressure if domestic work responsibilities are not shared between men and women. Therefore, increasing women’s economic power may not be enough to protect them from IPV and improve their mental health if societal attitudes and norms are ignored. Interestingly, an intervention combining economic empowerment with social norms transformations led to a significant reduction in IPV for women in some countries from the Global South, such as Uganda, Kenia, Peru, and Afghanistán (Kerr-Wilson et al., 2020).

Furthermore, we explored whether the impact of *power to* on IPV and mental health was explained by the agency. The results showed that women’s agency was a potential mechanism through which power could reduce IPV and improve women’s mental health. Specifically, we found that education had an empowering effect and increased both intrinsic and instrumental agency, but only instrumental agency negatively predicted the probability of being a victim of IPV.

However, economic power did not improve women’s instrumental agency. These results partially differ from those found in a review by Brody et al. (2015) in which endowing women with economic resources had an impact on economic and political empowerment, decision-making and mobility outside the family, as well as social empowerment. In our study, there was no significant impact of economic power on the intrinsic agency. Yount et al. (2021) found that economic empowerment increased women’s autonomy, but similarly, neither any significant differences were found in their awareness of rights, nor in their attitudes toward gender inequality and justifications of violence against women (defined in our study as intrinsic agency). The authors argue that women may be conforming not only to community norms based on a long patriarchal history, but also that their initiative was not designed to sufficiently influence norms changes. Indeed, others have found that women’s psychological empowerment improved as they gained a public voice and were respected by community members, enhancing the quality of their social networks and solidarity among the community members (Brody et al., 2015). These results appear plausible given the pervasiveness of sexism and patriarchal structures across all areas of society and given that IPV can affect women of any social class or educational level.

Finally, the positive effect of *power to* (education) on mental health was partially mediated by the intrinsic agency. That is, *power to* (education) increased women’s instrumental agency, which in turn reduced their likelihood of suffering IPV, and such a reduction improved their mental health. Therefore, agency (both intrinsic and instrumental) appeared to be a potential mechanisms (either on its own or through the prevention of IPV) for reducing women’s psychological distress. That is, social support, participation in community social networks, making decisions about one’s own reproductive health, and a greater consciousness of gender rights seemed to be protective factor for women’s mental health and wellbeing. Several studies in countries in the Global South support this result. For example, research shows that an increase in women’s intrinsic agency (agency regarding their attitudes about gender norms) reduced mental stress, whereas domains measuring instrumental agency (i.e., household decision-making; freedom of movement) did not (Richardson et al., 2019). Indeed, this domain of agency could be influenced by relational power dynamics (e.g., *power over*) which were not examined in this study but should be accounted for in future research. Another study conducted in India showed that lower levels of autonomy were associated with a higher probability of having a common mental disorder (Patel et al., 2006). Furthermore, freedom of movement was associated with lower levels of depressive symptoms and a decrease in systolic blood pressure among women in Uzbekistan (Hadley et al., 2010). Agency has been measured in very different ways, thus making comparisons across studies difficult and results are highly dependent upon the context in which women live (see Richardson et al., 2019).

The positive influence of education as opposed to economic resources on empowerment, protection against IPV, and mental health problems may be related to the socio-economic conditions of Salvadoran women. In El Salvador, women have more jobs in the informal sector than men; they earn less than men, suffer more vulnerable conditions and have been denied access to social security. In fact, in 31.1% of families, women are solely responsible for raising children and supporting the home. Furthermore, women’s wages are on average 15.5% lower than that of men, which reduces their development opportunities (United Nations Development Programme [UNDP], 2020a). Therefore, working and achieving one’s own economic resources does not guarantee an expansion of choices and life options for women in El Salvador. Our results suggest that power based on education protects women in El Salvador; one reason may be that they can then work in more qualified jobs and thus increase their life choices and opportunities. At the same time, increased social support, decision-making, and freedom of movement provide greater autonomy and are protective factors against IPV. All these indicators are related to suffering less control by men. Controlling violence is one of the most prevalent types of violence in El Salvador: about 41.2% of Salvadoran women suffer from controlling violence from their partners (Navarro-Mantas et al., 2021b). Social control over women is also common. For instance, El Salvador is one of the few countries in the world that criminalizes women for the termination of pregnancy: there are women serving sentences in prison for elective and non-elective abortions with the approval from most of the population (United Nations [UN], 2022). Controlling violence is one of the most accepted forms of violence because it is part of the culture. The idealized romantic relationship and gender roles are permeated with beliefs that favor women being controlled (Hume, 2008). Therefore, to design prevention policies of violence against women in El Salvador, it would be recommended to raise awareness about this type of controlling violence and promote messages about women’s autonomy and freedom to decide about their own lives, health, and bodies.

### Limitations and Future Research

Our study offers new insights into the link between power, agency, IPV, and mental health, but we acknowledge that the present study has two main limitations. First, it is important to note that we selected our indicators assessing *power to post hoc*. Future studies should employ a more suitable direct indicator of power, such as a scale designed to assess the underlying conceptual dimensions of gendered power (refer also Grose and Grabe, 2014; Richardson, 2018). Unfortunately, we were not able to include measures of *power over* as defined by previous literature (i.e., control over an intimate partner’s behavior) in our analyses as the data set did not include any suitable indicators. Future studies should include culturally sensitive measures of *power to* and *power over*. This would offer relevant information about women’s power with respect to their partners and how this influences women’s agency. Similarly, we selected and created the agency measures *post hoc* based on a theory making comparisons with other studies difficult. We used the standard methodology of the national surveys of WHO on violence against women, whose objective is to study the prevalence of violence against women and women’s health. The WHO survey did not include a validated scale to measure agency. For this reason, we used several survey items as proxy indicators to represent the construct of agency. Our research suggests the importance of including an index on power and agency in future national surveys, as it can be very informative to develop efficacious interventions and prevention programs by addressing not only women’s actual resources but their ability to make decisions and their critical thinking about gender relations. Second, the cross-sectional nature of the data does not allow us to draw any causal conclusions. This also influences the directionality of effects. For example, there could be a bidirectional (or reversed) relationship between some of the included variables, such that women who are subjected to IPV might be less likely to have agency in making reproductive decisions. However, we relied on theoretical (e.g., agency is a process) and applied assumptions (i.e., the nature of some of the variables suggests that they precede others in chronological terms). For example, the level of studies (education-based *power to*) refers to past achievements that can impact women’s agency (instrumental and intrinsic) and precedes participants’ experiences of IPV as operationalized in this study. Also, we looked at the association between violence experienced over one’s lifetime and their recent mental health symptoms (from the last month before the interview). Thus, we relied on our theoretical argumentation of the proposed predictive relationships between variables, but we have been cautious when drawing final conclusions based upon them. Future studies should investigate the theorized causal links between the variables longitudinally in El Salvador and other cultural contexts to offer new insights into how gendered power may increase or protect women against the experience of IPV.

### Practical Implications

There is a lot of discussion about the best ways of empowering women, particularly those living in the Global South (e.g., Jewkes, 2002; Lwamba et al., 2021). Microfinance services and cash transfer programs have proliferated in recent decades, especially for women in low-income countries, with the belief that economic empowerment increases women’s autonomy and power, and therefore protects them from violence (for an overview of microfinance services see Hansen et al., 2021). However, the evidence increasingly questions the transformative capacity of these programs without addressing the social and gender norms in the cultural context in which women live (Goetz and Gupta, 1996; Hansen et al., 2021; Lwamba et al., 2021; Yount et al., 2021). Furthermore, a purely economic approach in the Global South may also potentially cause harm as it may reproduce historical and ongoing forms of (neo)colonial domination (Kurtiş et al., 2016). It may stimulate a change in the social fabric of the communities by introducing the concept of market pricing in communities that were still strongly based on communal sharing (Fiske, 1992). This may not help to promote the sustainable development of the communities in general.

This study offers three important practical implications for governments and societal actors who strive to design interventions, programs, and public policies aimed at preventing IPV and mental health problems for women in the Global South.

First, as also suggested by others, education is a key for strengthening women’s position in society (Ludermir et al., 2008; Ishida et al., 2010; Navarro-Mantas et al., 2021a). Our results suggest that greater educational power is also linked to higher levels of agency, which should help women to develop a greater ability to control their life options, make their own decisions, and improve their mental health. Education offers women more qualifications, which in turn provides them with better options for a better quality of life, expanded social networks, and greater autonomy. Girls in El Salvador stay in school on average for 6.6 years, compared to boys who stay longer for 7.3 years on average (United Nations Development Programme [UNDP], 2020b). Thus, campaigns and initiatives focusing on helping girls to remain in school could be effective in increasing the mental health of young women and reducing their likelihood of being subjected to IPV in the long run.

Second, with respect to employment, our data indirectly stresses the importance of influencing other structures to change gender inequity. More precisely, developing programs or policies which could help to decrease/end informal work for women to create more formal employment options would normalize women working and be intervening in the feminization of poverty. This may be a more effective strategy to invoke change at a societal level and alter prevailing traditional gender norms (for similar arguments see Goetz and Gupta, 1996; Grose and Grabe, 2014).

Third, change toward more gender justice requires an approach that involves men as well as women (e.g., Rahman et al., 2011; Hansen et al., 2021). For example, men who participated in programs about economic empowerment and norm transformation, reported inflicting significantly less physical and financial IPV, as well as a reduction in sexual IPV, and sexual violence outside of their relationship (Gibbs et al., 2020). Educating men implies questioning the gender structure and the legitimacy of patriarchal privilege and the hegemony of masculine norms to achieve more gender-equal attitudes and prevent IPV (Abramsky et al., 2014; Gibbs et al., 2018). Other studies show that men and women both need to be involved when deciding about family planning to change existing patterns while preserving women’s autonomy (Petterson and Sutton, 2018). Thus, programs that involve men and women might be promising avenues to achieve more gender justice.

### Conclusion

One of the sustainable development goals is achieving gender equity and empowering all women and girls, especially in countries in the Global South (UN General Assembly, 2015). The first step to achieving these objectives is ending the violence that millions of women and girls suffer daily around the world. The majority of intervention studies have focused on women’s economic empowerment to prevent violence and improve their mental health. However, our results suggest that other factors might be more influential in protecting women. Supporting women to develop stronger agency, that is, their own opportunities, autonomy, and decisions for the future seems to be a very promising way. Our study suggests that promoting interventions that further increase women’s empowerment (through promoting their agency, personal qualifications, and access to life with greater gender equality) and access to education as well as targeting traditional gender norms may help to decrease and hopefully prevent IPV and improve the well-being and gender justice more generally for women in El Salvador and other nations in the Global South.

## Data Availability Statement

The original contributions presented in the study are included in the article/Supplementary Material, further inquiries can be directed to the corresponding author.

## Ethics Statement

The studies involving human participants were reviewed and approved by Ethics Review Committee of the Pan American Health Organization (PAHOERC). Written informed consent to participate in this study was provided by the participants’ legal guardian/next of kin.

## Author Contributions

LN-M, SL, LM, NH, and JM contributed to the discussion and development of the conceptual framework of this manuscript, conducted the literature review, and contributed to the writing of the full manuscript. LN-M prepared and conducted the fieldwork. LM conducted preliminary analyses as part of her master thesis. EG-S analyzed the data and reported the method and results sections. LN-M, EG-S, and LM wrote the first draft. All authors approved the final version of the manuscript.

## Conflict of Interest

The authors declare that the research was conducted in the absence of any commercial or financial relationships that could be construed as a potential conflict of interest.

## Publisher’s Note

All claims expressed in this article are solely those of the authors and do not necessarily represent those of their affiliated organizations, or those of the publisher, the editors and the reviewers. Any product that may be evaluated in this article, or claim that may be made by its manufacturer, is not guaranteed or endorsed by the publisher.

## Footnotes

1 It is important to keep in mind that the diversity of reported findings may in part also be explained by two main methodological differences in the studied interventions; (1) diversity of studied services (e.g., credit, training, individual vs. group lending models) and (2) diversity of study designs (e.g., nationwide survey data, randomized control trials, and qualitative research).
