# Supplementary material for: Defining Power and Agency in Gender Relations in El Salvador: Consequences for Intimate Partner Violence and Women’s Mental Health
Source: Front Psychol. 2022 Apr 19;13:867945. doi: 10.3389/fpsyg.2022.867945 (PMC9063008; doi:10.3389/fpsyg.2022.867945)
Supplement: Supplementary file 1 [file Table_1.DOCX]

Supplementary Material:

**Defining power and agency in gender relations in El Salvador: Consequences for intimate partner violence and women’s mental health**

**Laura Navarro-Mantas^1*^, Soledad de Lemus^2^, Efraín García-Sánchez^2^, Lucy McGill^3^, Nina Hansen^3^, & Jesús L. Megías^2^**

^1^ International University of La Rioja (UNIR), Faculty of Education, Rioja, Spain

^2^ University of Granada, Mind, Brain and Behavior Research Center at University of Granada (CIMCYC-UGR), Granada, Spain

^3^ University of Groningen, Institute of Psychology, Social Psychology Department, Groningen, The Netherlands

***Correspondence:**Laura Navarro-Mantas, [laura.navarro@unir.net](mailto:laura.navarro@unir.net)

**Contenido**

[Table S1. Descriptive statistics of the indicators used to compute the variables included in the study 2](#_Toc94635477)

[Table S2. Standardized coefficients for the measurement and structural model implied in Model 1. 4](#_Toc94635478)

[Table S3. Standardized coefficients for the measurement and structural model implied in Model 2. 5](#_Toc94635479)

[Table S4. Standardized coefficients for the measurement and structural model implied in Model 3. 6](#_Toc94635480)

| Table S1. Descriptive statistics of the indicators used to compute the variables included in the study | | | | | |
| --- | --- | --- | --- | --- | --- |
| Variable | Indicator | N | M | SD | Range |
| Mental health |  |  |  |  |  |
|  | 209p. Do you feel that you are a worthless person ? | 1085 | 0.93 | 0.25 | 1 (0-1) |
|  | 209l. a)     Do you find it difficult to make decisions? | 1086 | 0.79 | 0.4 | 1 (0-1) |
|  | 209n. Do you feel unable to play a useful role in life? | 1085 | 0.9 | 0.3 | 1 (0-1) |
|  | 209k. Do you find it difficult to enjoy your daily activities? | 1085 | 0.84 | 0.37 | 1 (0-1) |
|  | 209o. Have you lost interest in things? | 1086 | 0.89 | 0.31 | 1 (0-1) |
|  | 209m. Do you find it difficult to do daily work? | 1088 | 0.86 | 0.34 | 1 (0-1) |
|  | 209h. Do you have problems thinking clearly? | 1086 | 0.86 | 0.35 | 1 (0-1) |
|  | 209j. Are you crying more than usual? | 1087 | 0.78 | 0.41 | 1 (0-1) |
|  | 209q. Have you thought about ​​ending your life? | 1085 | 0.94 | 0.24 | 1 (0-1) |
| IPV (sexual) |  |  |  |  |  |
|  | 706A.A.A. Has your current husband / partner or another partner forced you to have sexual intercourse by threatening, holding you down or harming you in any way? | 1081 | 0.1 | 0.3 | 1 (0-1) |
|  | 706A.B. Did you ever agree to have sexual intercourse when you did not want to and you did it because you were afraid of what your husband / partner might do if you refused? | 1079 | 0.11 | 0.31 | 1 (0-1) |
|  | 706A.C. Has your current partner or another partner ever forced you to perform any sexual act (other than vaginal intercourse) that you did not want to do? | 1078 | 0.06 | 0.23 | 1 (0-1) |
| IPV (physical) | |  |  |  |  |
|  | Has your current partner or another partner ever:  705A.A.A. Slapped you or thrown things that could hurt you? | 1078 | 0.17 | 0.38 | 1 (0-1) |
|  | 705A.B. Pushed you, cornered you or pulled your hair? | 1077 | 0.19 | 0.39 | 1 (0-1) |
|  | 705A.C. Hit you with his fist, with a Corvo knife or with something else that could hurt you? | 1077 | 0.13 | 0.34 | 1 (0-1) |
|  | 705A.D. Kicked you, dragged you or beaten you up? | 1077 | 0.09 | 0.28 | 1 (0-1) |
|  | 705a.e. Tried to strangle you or burn you on purpose? | 1075 | 0.06 | 0.24 | 1 (0-1) |
|  | 705A.F. Threatened to use or actually used a gun, knife or other weapon against you? | 1076 | 0.07 | 0.25 | 1 (0-1) |
| IPV (control) |  |  |  |  |  |
|  | When thinking about your (current/most recent) husband/partner or another husband/partner you have had in the past, would you say it is/was generally true that he:  703A.A. Tries to prevent you from seeing your friends? | 1081 | 0.19 | 0.39 | 1 (0-1) |
|  | 703A.C. Insists on knowing where you are at all times? | 1082 | 0.25 | 0.43 | 1 (0-1) |
|  | 703A.F. Often suspects that you are unfaithful? | 1080 | 0.13 | 0.34 | 1 (0-1) |
|  | 703A.H. Refuses or refused to give you enough money for household expenses, even when he has money for other things? | 1077 | 0.13 | 0.34 | 1 (0-1) |
|  | 703A.J. Prevent or make it difficult for you to participate in meetings with other people, in the community or in the church? | 1081 | 0.1 | 0.3 | 1 (0-1) |
| IPV (psychological) | |  |  |  |  |
|  | Think about your current husband/partner, or any other partner:  704a.a.a. Does he ever insult you or make you feel bad about yourself? | 1082 | 0.35 | 0.48 | 1 (0-1) |
|  | 704a.b. Does he ever belittle or humiliate you in front of other people? | 1081 | 0.22 | 0.41 | 1 (0-1) |
|  | 704a.c. Does he ever do things to scare or intimidate you on purpose (for example, by the way he looks at you, by yelling or breaking things)? | 1080 | 0.15 | 0.35 | 1 (0-1) |
|  | 704a.d. Does he ever verbally threaten to hit or hurt you or someone important to you? | 1080 | 0.16 | 0.37 | 1 (0-1) |
|  | 704a.e. Does he ever not talk to you or act as if you do not exist? | 1075 | 0.16 | 0.37 | 1 (0-1) |
|  | 704A.F. Does he ever harass you by sending messages, making phone calls ...? | 1071 | 0.07 | 0.26 | 1 (0-1) |
| *(Continued)* | | | | | |

| Table S1. (Continued) | | | | | |
| --- | --- | --- | --- | --- | --- |
| Variable | Indicator | N | M | SD | Range |
| Power to (economic) | |  |  |  |  |
|  | 111c. What is your main daily occupation? | 1085 | 0.29 | 0.45 | 1 (0-1) |
|  | 1102.A. Do you do anything to earn money? | 1074 | 0.52 | 0.5 | 1 (0-1) |
|  | Do you own any of the following things alone or with another person:  1101a. Land. | 1070 | 0.16 | 0.37 | 1 (0-1) |
|  | 1101b. House. | 1076 | 0.36 | 0.48 | 1 (0-1) |
|  | 1101c. A company or business. | 1065 | 0.06 | 0.24 | 1 (0-1) |
|  | 1101j. Car. | 1068 | 0.1 | 0.3 | 1 (0-1) |
| Power to (education) | |  |  |  |  |
|  | 111a. What is the highest level of education that you have completed? | 1078 | 1.49 | 0.86 | 3 (0-3) |
| Instrumental agency | |  |  |  |  |
|  | 118a. Have you ever been prevented from attending a meeting or participating in an organization? | 1084 | 0.96 | 0.2 | 1 (0-1) |
|  | 1105. Have you ever left / rejected a job because your husband/partner did not want you to work? | 1068 | 0.6 | 0.49 | 1 (0-1) |
|  | 114. How often do you see or talk to someone from your birth family? At least once a week, once a month, once a year or never? | 1029 | 0.91 | 0.28 | 1 (0-1) |
|  | 115. When you need help or have a problem, can you generally count on members of your birth family for support? | 1082 | 0.89 | 0.32 | 1 (0-1) |
|  | 311. Have you ever taken care of or used anything to delay or prevent pregnancy? | 1052 | 0.48 | 0.5 | 1 (0-1) |
|  | 318. Have you ever asked your current/most recent husband/partner to use a condom? | 1037 | 0.2 | 0.4 | 1 (0-1) |
| Intrinsic agency | |  |  |  |  |
|  | Do you agree or disagree with the following statements:  601. A good wife obeys her husband even if she does not agree with him | 1065 | 0.67 | 0.47 | 1 (0-1) |
|  | 602. It is important for a man to show his wife / partner who is the boss | 1072 | 0.69 | 0.46 | 1 (0-1) |
|  | 603. A woman should be able to choose her own friends even if her husband does not agree | 1059 | 0.82 | 0.38 | 1 (0-1) |
|  | 604. It is the wife’s obligation to have sexual relations with her husband even if she does not want to ? | 1065 | 0.91 | 0.29 | 1 (0-1) |
| Covariates |  |  |  |  |  |
|  | 107. How old were you on your last birthday? | 1089 | 38.27 | 13.14 | 49 (15-64) |
|  | Zone (urban/rural). | 1087 | 0.35 | 0.48 | 1 (0-1) |
|  | 303. How many children do you have that are alive today? | 934 | 2.67 | 1.61 | 14 (1-15) |
| Note: M = Mean; IPV = Intimate Partner violence | |  |  |  |  |

| Table S2. Standardized coefficients for the measurement and structural model implied in Model 1. | | | | |
| --- | --- | --- | --- | --- |
| **Measurement model** |  |  |  |  |
| Latent variable | Indicators | B | SE (std.) | 95% CI |
| Intrinsic agency | Autonomy | 0.872*** | 0.052 | [0.770, 0.973] |
|  | Power | 0.748*** | 0.051 | [0.649, 0.847] |
|  | Frienship election | 0.344*** | 0.061 | [0.224, 0.465] |
|  | Sexual independence | 0.662*** | 0.061 | [0.542, 0.781] |
| IPV (sexual) | Forced | 0.981*** | 0.012 | [0.957, 1.005] |
|  | Unconsented | 0.943*** | 0.016 | [0.913, 0.974] |
|  | Unwanted practice | 0.956*** | 0.023 | [0.910, 1.001] |
| IPV (physical) | Throw things | 0.973*** | 0.009 | [0.956, 0.990] |
|  | Pushed | 0.946*** | 0.012 | [0.922, 0.970] |
|  | Hit | 0.966*** | 0.011 | [0.946, 0.987] |
|  | Kick | 0.968*** | 0.012 | [0.945, 0.992] |
|  | Strangle | 0.959*** | 0.013 | [0.935, 0.984] |
|  | Threat | 0.987*** | 0.009 | [0.970, 1.005] |
| IPV (control | Prevent friends | 0.866*** | 0.025 | [0.817, 0.915] |
|  | Harrassed | 0.847*** | 0.024 | [0.799, 0.894] |
|  | Unfaithful | 0.856*** | 0.030 | [0.798, 0.914] |
|  | Money | 0.858*** | 0.030 | [0.798, 0.917] |
|  | Isolating | 0.931*** | 0.026 | [0.880, 0.982] |
| IPV (psychological) | Insult | 0.957*** | 0.014 | [0.929, 0.985] |
|  | Humilliate | 0.920*** | 0.017 | [0.887, 0.952] |
|  | Intimidate | 0.940*** | 0.014 | [0.912, 0.968] |
|  | Threat | 0.954*** | 0.013 | [0.929, 0.979] |
|  | Ignore | 0.873*** | 0.021 | [0.831, 0.915] |
|  | Chasing | 0.874*** | 0.026 | [0.823, 0.926] |
| IPV (second order latent variable) | Sexual | 0.922*** | 0.018 | [0.888, 0.957] |
|  | Physical | 0.927*** | 0.016 | [0.896, 0.958] |
|  | Control | 0.833*** | 0.027 | [0.780, 0.887] |
|  | Psychological | 0.966*** | 0.013 | [0.940, 0.992] |
| Mental health | Whorthless | 0.898*** | 0.034 | [0.832, 0.964] |
|  | Decision making | 0.802*** | 0.032 | [0.738, 0.865] |
|  | Incapable | 0.783*** | 0.039 | [0.708, 0.859] |
|  | Disfunctional | 0.833*** | 0.031 | [0.772, 0.893] |
|  | Uninterested | 0.855*** | 0.030 | [0.796, 0.914] |
|  | Working issues | 0.676*** | 0.048 | [0.583, 0.769] |
|  | Thinking | 0.713*** | 0.043 | [0.630, 0.797] |
|  | Crying | 0.737*** | 0.042 | [0.655, 0.820] |
|  | Suicidal thoughts | 0.735*** | 0.063 | [0.610, 0.859] |
| **Regression coefficients** |  |  |  |  |
| Outcome (endogenous variable) | Indicators (exogenous variable) | B | SE (std.) | 95% CI |
| IPV (total) | Power to (economic) | 0.207*** | 0.043 | [0.124, 0.290] |
|  | Power to (social) | -0.123** | 0.047 | [-0.214, -0.031] |
|  | Rural | -0.062 | 0.044 | [-0.149, 0.024] |
|  | Rural (vs. urban) | 0.075 | 0.048 | [-0.019, 0.169] |
|  | Age | 0.056 | 0.049 | [-0.039, 0.152] |
|  | Power to (economic) | 0.155*** | 0.046 | [0.064, 0.246] |
|  | Power to (social) | 0.161*** | 0.046 | [0.072, 0.251] |
|  | IPV (total) | -0.367*** | 0.051 | [-0.468, -0.267] |
|  | Rural | -0.055 | 0.043 | [-0.140, 0.030] |
|  | Rural (vs. urban) | -0.075 | 0.044 | [-0.162, 0.012] |
|  | Age | 0.073 | 0.049 | [-0.023, 0.169] |

| **Table S3.** Standardized coefficients for the measurement and structural model implied in Model 2. | | | | |
| --- | --- | --- | --- | --- |
| Measurement model |  |  |  |  |
| Latent variable | Indicators | B | SE (std.) | 95% CI |
| Intrinsic agency | Autonomy | 0.842*** | 0.048 | [0.747, 0.937] |
|  | Power | 0.824*** | 0.050 | [0.725, 0.922] |
|  | Frienship election | 0.392*** | 0.060 | [0.274, 0.510] |
|  | Sexual independence | 0.674*** | 0.055 | [0.566, 0.782] |
| IPV (sexual) | Forced | 0.981*** | 0.012 | [0.957, 1.006] |
|  | Unconsented | 0.943*** | 0.016 | [0.912, 0.974] |
|  | Unwanted practice | 0.956*** | 0.023 | [0.911, 1.001] |
| IPV (physical) | Throw things | 0.972*** | 0.009 | [0.955, 0.990] |
|  | Pushed | 0.946*** | 0.013 | [0.921, 0.970] |
|  | Hit | 0.966*** | 0.011 | [0.946, 0.987] |
|  | Kick | 0.969*** | 0.012 | [0.945, 0.992] |
|  | Strangle | 0.959*** | 0.013 | [0.934, 0.984] |
|  | Threat | 0.987*** | 0.009 | [0.970, 1.005] |
| IPV (control | Prevent friends | 0.865*** | 0.025 | [0.816, 0.914] |
|  | Harrassed | 0.845*** | 0.024 | [0.798, 0.892] |
|  | Unfaithful | 0.856*** | 0.030 | [0.797, 0.914] |
|  | Money | 0.859*** | 0.030 | [0.800, 0.919] |
|  | Isolating | 0.932*** | 0.026 | [0.881, 0.983] |
| IPV (psychological) | Insult | 0.957*** | 0.014 | [0.929, 0.985] |
|  | Humilliate | 0.920*** | 0.017 | [0.887, 0.953] |
|  | Intimidate | 0.940*** | 0.014 | [0.912, 0.968] |
|  | Threat | 0.954*** | 0.013 | [0.928, 0.979] |
|  | Ignore | 0.873*** | 0.021 | [0.831, 0.915] |
|  | Chasing | 0.873*** | 0.027 | [0.821, 0.925] |
| IPV (second order latent variable) | Sexual | 0.921*** | 0.018 | [0.887, 0.956] |
|  | Physical | 0.926*** | 0.016 | [0.895, 0.957] |
|  | Control | 0.834*** | 0.027 | [0.780, 0.887] |
|  | Psychological | 0.966*** | 0.013 | [0.940, 0.992] |
| Mental health | Whorthless | 0.900*** | 0.033 | [0.834, 0.965] |
|  | Decision making | 0.803*** | 0.032 | [0.740, 0.867] |
|  | Incapable | 0.785*** | 0.039 | [0.709, 0.860] |
|  | Disfunctional | 0.831*** | 0.031 | [0.770, 0.892] |
|  | Uninterested | 0.857*** | 0.030 | [0.798, 0.915] |
|  | Working issues | 0.675*** | 0.048 | [0.582, 0.768] |
|  | Thinking | 0.715*** | 0.043 | [0.632, 0.799] |
|  | Crying | 0.732*** | 0.042 | [0.649, 0.814] |
|  | Suicidal thoughts | 0.733*** | 0.064 | [0.607, 0.858] |
| (*continued*) | | | | |

| Table S3. (*continued*) | | | | |
| --- | --- | --- | --- | --- |
| Structural model |  |  |  |  |
| **Regression coefficients** |  |  |  |  |
| Outcome (endogenous variable) | Indicators (exogenous variable) | B | SE (std.) | 95% CI |
| Intrinsic agency | Power to (economic) | 0.086 | 0.047 | [-0.007, 0.178] |
|  | Power to (education) | 0.299*** | 0.046 | [0.209, 0.388] |
|  | Rural (vs. urban) | -0.116** | 0.045 | [-0.203, -0.028] |
|  | Number of living children | -0.017 | 0.051 | [-0.116, 0.082] |
|  | Age | -0.062 | 0.057 | [-0.173, 0.049] |
| Instrumental agency | Power to (economic) | 0.015 | 0.031 | [-0.045, 0.076] |
|  | Power to (education) | 0.085* | 0.037 | [0.014, 0.157] |
|  | Rural (vs. urban) | -0.077* | 0.034 | [-0.145, -0.010] |
|  | Number of living children | -0.011 | 0.041 | [-0.091, 0.070] |
|  | Age | -0.329*** | 0.053 | [-0.433, -0.225] |
| IPV (total) | Power to (economic) | 0.200*** | 0.042 | [0.118, 0.282] |
|  | Power to (education) | -0.136** | 0.050 | [-0.234, -0.037] |
|  | Intrinsic agency | 0.110 | 0.060 | [-0.008, 0.228] |
|  | Instrumental agency | -0.178*** | 0.040 | [-0.256, -0.099] |
|  | Rural (vs. urban) | -0.062 | 0.044 | [-0.148, 0.024] |
|  | Number of living children | 0.072 | 0.047 | [-0.021, 0.165] |
| Mental health | Power to (economic) | 0.139** | 0.046 | [0.049, 0.229] |
|  | Power to (education) | 0.096* | 0.048 | [0.001, 0.191] |
|  | Intrinsic agency | 0.183*** | 0.055 | [0.075, 0.290] |
|  | Instrumental agency | 0.102* | 0.040 | [0.023, 0.180] |
|  | IPV | -0.370*** | 0.051 | [-0.470, -0.269] |
|  | Rural (vs. urban) | -0.026 | 0.043 | [-0.111, 0.059] |
|  | Number of living children | -0.071 | 0.044 | [-0.158, 0.016] |
|  | Age | 0.115* | 0.052 | [0.014, 0.216] |
